# Selection of Potential Potato Cultivars With Export Quality Through Multitrait Genotype‐Ideotype Distance Index (MGIDI)

**DOI:** 10.1002/fsn3.71524

**Published:** 2026-02-10

**Authors:** Md Mazadul Islam, Md Nurul Amin, Md Mushfiqur Rahman, Sauda Naznin, Md Nasir Uddin, Afroz Naznin, Tuhina Hasan, Md Salim, Md Babul Anwar, Abdulrahman Alasmari, Ahmed Gaber, Mohammed M. Althaqafi, Akbar Hossain

**Affiliations:** ^1^ Tuber Crops Research Centre Bangladesh Agricultural Research Institute Gazipur Bangladesh; ^2^ Department of Biotechnology Bangladesh Agricultural Research Institute Gazipur Bangladesh; ^3^ Horticulture Research Centre Bangladesh Agricultural Research Institute Gazipur Bangladesh; ^4^ Tuber Crops Research Sub Centre Bangladesh Agricultural Research Institute Munshiganj Bangladesh; ^5^ Regional Agricultural Research Station Bangladesh Agricultural Research Institute Jashore Bangladesh; ^6^ Department of Biology, Faculty of Science University of Tabuk Tabuk Saudi Arabia; ^7^ Department of Biology, Faculty of Science Taif University Taif Saudi Arabia; ^8^ Department of Biotechnology, College of Sciences Taif University Taif Saudi Arabia; ^9^ Soil Science Division Bangladesh Wheat and Maize Research Institute Dinajpur Bangladesh

**Keywords:** MGIDI, multitrait selection, potato breeding, potato export, yield

## Abstract

Potato is a strategic crop that contributes significantly to food security due to its short growing period and high yield per unit area. Genotype selection based on multiple traits is important for identifying superior germplasm. The goal of this study was to identify promising potato genotypes with export potential to enhance potato exports from Bangladesh. This study was conducted across four agroecological zones in Bangladesh during 2019–2020 and 2020–2021 to identify high‐quality potato varieties suitable for export using the multitrait genotype‐ideotype distance index (MGIDI). Thirty potato varieties released by the Bangladesh Agricultural Research Institute (BARI) were evaluated for traits such as plant height, stem number per hill, tuber weight per hill, tuber yield, dry matter, tuber grading, storability, and disease incidence. MGIDI analysis identified six superior genotypes viz., BARI Alu‐7, BARI Alu‐40, BARI Alu‐46, BARI Alu‐97, BARI Alu‐62, and BARI Alu‐37 considering a selection intensity of 20%. These genotypes demonstrated strong performance, revealing both their strengths and weaknesses in relation to the identified factors. Considering export features, BARI Alu‐62 and BARI Alu‐97 were considered the best among those six varieties for producing export quality potato. This research reveals the complex relationship between potato traits, showing how multitrait genotype selection can guide breeding strategies. Enhancing these traits can boost Bangladesh's potato exports, benefiting the economy and society.

## Graphical abstract

Ranking and selection of potato varieties using the MGIDI index. MGIDI analysis identified six superior genotypes viz., BARI Alu‐7, BARI Alu‐40, BARI Alu‐46, BARI Alu‐97, BARI Alu‐62, and BARI Alu‐37 considering a selection intensity of 20%. These genotypes demonstrated strong performance, revealing both their strengths and weaknesses in relation to the identified factors. Considering export features BARI Alu‐62 and BARI Alu‐97 considered the best among those selected six varieties for producing export quality potato. This research reveals the complex relationship between potato traits, showing how multitrait genotype selection can guide breeding strategies.

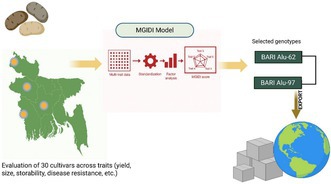

## 1 Introduction

Potatoes stand out as a crucial food crop on a global scale that contribute to global food security and are regularly consumed by billions of people around the world. The potato is highly valued for its ability to mature quickly during times of urgent food demand, making it a crucial commodity for ensuring food security and alleviating hunger (Amin et al. 2023; Hassen et al. 2015). Two advantageous characteristics of potato for food security are its optimal maturation period and high yield per unit area (Chala and Dechasa 2015; Girma et al. 2004). The increasing prevalence of hunger globally will result in a significant issue of chronic food insecurity during the 21st century. Hence, the cultivation of potatoes holds great potential in addressing global food needs, combating hunger, and alleviating poverty (Amin et al. 2023). Potato is highly nutritious and contains a significant amount of starch and minimal levels of sugar and fat. The product is rich in essential vitamins such as vitamin B6, niacin, folate, and vitamin C. It also contains important minerals like potassium, iron, phosphorus, and magnesium (Galdón et al. 2012; Gibson and Kurilich 2013). Additionally, it provides a substantial quantity of antioxidants, essential for modulating blood cholesterol levels (Amin et al. 2022).

Worldwide potato production in 2022 was 374.77 million tonnes, with China leading the way, followed by India, Ukraine, Russia, and the United States (FAOSTAT 2022). In recent decades, Bangladesh has witnessed substantial growth in the land area allocated for potato farming, alongside increased production and yield. Bangladesh is ranked 7th globally in terms of potato production, making it one of the leading potato‐producing countries. Moreover, the potato is acknowledged as the second most significant food crop in the nation. The nation's mean potato yield is 21.86 metric tonnes per hectare (t ha^−1^), resulting in a total production of 10.14 million metric tonnes (Mt) from a land area of 0.46 million hectares (Mha) (FAOSTAT 2022). This is a 1.8‐fold increase in production per unit area over the few decades (Naznin et al. 2023). Notably, only 66% of the total potato production is consumed as table potatoes, while a small fraction is allocated for processing purposes, leaving the majority of potatoes in surplus inside the country (Islam, Uddin, et al. 2022). Thus, utilizing the surplus potatoes for industrial processing or exporting them to international markets presents a practical and viable solution. Potato exports from Bangladesh hold the promise of reaching over 30 countries, facilitated by the participation of 60 companies in the industry (Islam, Uddin, et al. 2022). During the year 2014–2015, the quantity of exported potatoes was around 100,000 tons. However, this quantity has declined since then. For growers aiming to market their high‐quality produce, selecting the appropriate variety is pivotal to enhancing their export potential (Mohammadi et al. 2010). Farmers require cultivars that yield significantly in various environmental conditions and across extended periods. Given the large financial outlays for fertilizers and plant protection chemicals, it is imperative to safeguard farmers' interests by ensuring that potato harvests have predictable genotype yields.

The export potato variety possesses specific criteria that differ from those of our normal table potato type. For export, it is recommended to choose a potato variety characterized by high yield, size ≥ 40 mm, > 60% of tubers in the 41–55 mm grade, high dry matter content (≥ 19%), long storage capacity (≤ 5% weight loss), and shiny color. The Tuber Crops Research Centre (TCRC) of the Bangladesh Agricultural Research Institute (BARI) is actively involved in developing potato varieties with a wide range of desirable traits. As of 2021, BARI has successfully introduced a total of 100 distinct potato varieties (BARI 2021). So far, the different types of potatoes have not been tested specifically for their ability to be exported in different agroecological conditions. Evaluating such varieties for their export attributes such as high yield, dry matter, greater size, brilliant color, strong storage capacity, and disease resistance will be a thrilling endeavor. This evaluation has the potential to significantly impact the potato export industry in Bangladesh. Bangladesh has a lot of prospects in this area because of the substantial potential for exporting potatoes to nearby nations like Nepal, Sri Lanka, and Bhutan. Bangladeshi exporters should target the markets of Gulf countries, Malaysia, Dubai, and the Middle East, as the country has the capacity to manufacture high‐quality potatoes and products for export at a lower cost. Exporting potatoes is a significant means to increase the usage of domestically cultivated potatoes. Exporting potatoes not only contributes to the GDP but also brings about various socioeconomic advantages. These include creating more job opportunities, enhancing income and lifestyle, reducing rural‐to‐urban migration, and minimizing postharvest and storage losses. In this context, white‐skinned potato cultivars were prioritized in our study, as they are preferred in key export markets such as Malaysia, Sri Lanka, and the Middle East due to their uniform appearance, smooth texture, and higher consumer acceptability. Red‐skinned varieties were not included, as exporters reported lower demand for them in international shipments from Bangladesh.

The MGIDI is a unique approach developed for genotype selection by evaluating breeding values across many variables (Olivoto and Nardino 2021). Traditional ranking techniques, such as Fisher's least significant difference (LSD) and Tukey's honest significant difference (HSD) in ANOVA, evaluate genotypes based on a singular characteristic, whereas the MGIDI index assesses genotypes according to their performance across many traits. Furthermore, MGIDI resolves collinearity issues through the application of component analysis for indexing purposes. This methodology has been successfully employed in several crop breeding initiatives, including barley (Pour‐Aboughadareh and Poczai 2021), wheat (Lima et al. 2022), corn (Yue et al. 2022), rice (Debsharma et al. 2022), black oat (Klein et al. 2023), and maize (Singamsetti et al. 2023).

While our earlier study (Islam, Uddin, et al. 2022) focused on initial screening of export potential potato varieties within a single season, the current research significantly advances that effort by incorporating a multilocation, multiyear, and multitrait evaluation using the MGIDI index. This is the first systematic application of MGIDI in potato across diverse agroecological zones in Bangladesh, integrating eight export‐relevant traits over two consecutive years. Although MGIDI has been applied in other crops, its use for export‐oriented potato cultivar screening under real world, multi‐environment conditions is novel. This study uniquely integrates yield stability, quality, and storability traits, offering a comprehensive tool to align genotype performance with export market demands.

Given the foregoing context, the aim of this study is to use MGIDI index analysis to find premium potato varieties appropriate for export. Specifically, it addresses the following research questions: (1) Which BARI released potato cultivars meet key export trait thresholds, such as high yield, desirable tuber size, dry matter content, storability, and disease resistance? (2) Can the MGIDI index effectively rank genotypes across multiple traits like yield, quality, and storage under diverse agroecological conditions in Bangladesh? By answering these questions, the study seeks to enhance the identification of export‐ready cultivars and contribute to sustainable potato production and utilization strategies that support Bangladesh's growing potato export industry.

## 2 Materials and Methods

### 2.1 Study Site, Weather, and Soil Characteristics

**FIGURE 1:**
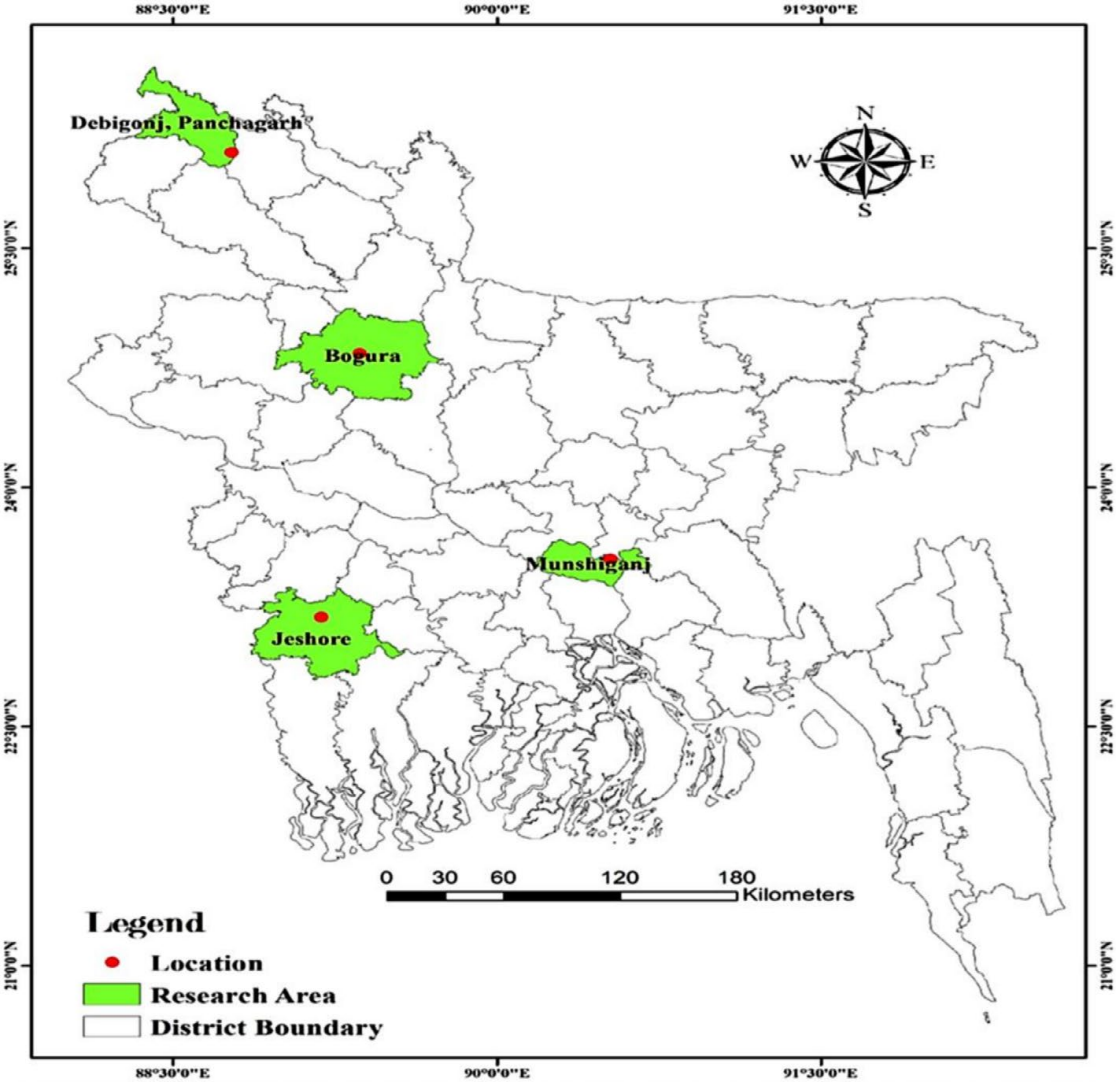
Locations of the study (Breeder seed production centre, BARI, Debiganj, Panchagarh, Bangladesh; Tuber Crops Research‐Sub Centre, BARI, Bogura, Bangladesh; and Tuber Crops Research‐Sub Centre, BARI, Munshigonj, Bangladesh).

**FIGURE 2:**
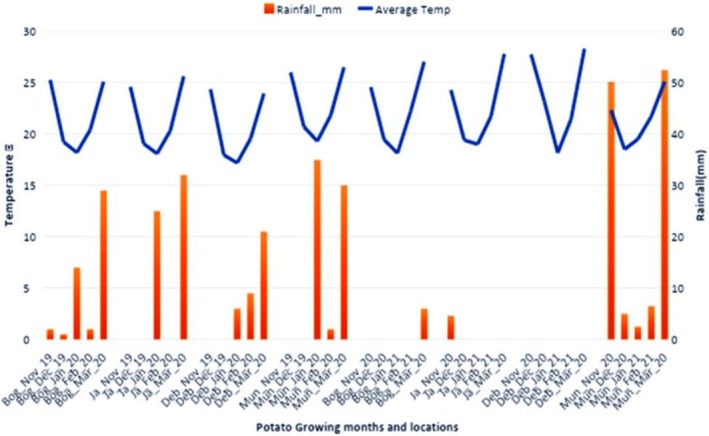
Average weather data for the potato cultivation period spanning (2019–2020 and 2020–2021) at four designated locations.

The field research was performed during the rabi season in two consecutive years (2019–2020 and 2020–2021) at four agroecological zones of Bangladesh. These sites were breeder seed production centre, BARI, Debiganj, Panchagarh (26°05′55.6″ N latitude and 88°46′11.5″ E longitude and 58 m above sea level), Regional Agricultural Research Station (RARS), BARI, Jashore (23°11′15.2″ N, 89°1′13.0″ E and 11 m above sea level), Tuber Crops Research‐Sub Centre (TCRSC), BARI, Bogura (24°50′27.1″ N, 89°21′51.9″ E and 22 m above sea level), and Tuber Crops Research‐Sub Centre (TCRSC), BARI, Munshigonj (23°3′08.8″ N, 90°31′27.7″ E and 8 m above sea level). The study sites are mapped in Figure 1, and climatic conditions are presented in Figure 2. The agroecological zones and soil parameters of the examined locations are presented in Table 1.

**TABLE 1:** Soil nutrient status and agroecological zones of four experimental sites.

Locations	Agro ecological zone (AEZ)	Soil texture	Soil organic matter	Nutrient status								
				N (%)	P (μg g^−1^)	K (mcq 100 g^−1^)	Ca (mcq 100 g^−1^)	Mg (mcq 100 g^−1^)	S (μg g^−1^)	Zn (μg g^−1^)	B (μg g^−1^)	Mo (μg g^−1^)
Debigonj, Panchagarh	AEZ 3: Tista Meander Floodplain	Sandy loam	Low medium	0–0.15	6.1–18	0.06–0.18	1.51–4.5	0.37–1.12	6.1–18	0.37–1.12	0.12–0.24	0.04–0.09
Jashore	AEZ 11: High Ganges River Floodplain	Sandy loam to silty loam	Low medium	0–0.15	0–12	0.06–0.18	4.51–7.5	1.12–1.87	6.1–18	0–0.75	0.12–0.24	0.09–0.13
Munshigonj	AEZ 19: Old Meghna Estuarine Floodplain	Sandy loam	Low medium	0–0.15	0–12	0.06–0.18	3.1–6	0.75–1.5	6.1–18	0.37–1.12	0.12–0.24	0.13–0.18
Bogura	AEZ 27: North‐Eastern Barind Tract	Loamy	Low medium	0–0.18	7.51–22.5	0.09–0.27	3.1–6	0.75–1.5	7.51–22.5	0.45–1.35	0.15–0.45	0.07–0.22

### 2.2 Experimental Materials and Design

The experimental material consisted of 30 white‐skinned BARI potato types. The tuber properties of the chosen cultivars are displayed in Table S1. The study was established as a randomized complete block (RCB) design and reproduced three times at each of the examined locations. Each trial was conducted in a plot measuring 6 m × 5 m, with planting occurring between 15 and 25 November in the years 2019–2020 and 2020–2021.

### 2.3 Experimental Techniques and Management

In the 2019–2020 and 2020–2021 trials, disease‐free, healthy whole seed tubers measuring 28–40 mm were planted with a 25 cm plant spacing and a 60 cm row spacing. A nutrient management protocol was implemented, incorporating organic manure (cow dung) and chemical fertilizers, including urea, muriate of potash, triple super phosphate, boric acid, zinc sulfate, and gypsum, as per the guidelines (Ahmmed et al. 2018). At planting, half of the urea and the full dosage of other fertilizers were applied in furrows positioned alongside the tuber rows. The remaining urea was top‐dressed during earthing up, conducted 30–35 days after planting (DAP). Routine intercultural practices, such as weeding and earthing up, were performed according to recommendations from the TCRC and BARI guidelines (Islam, Naznin, et al. 2022). Plant protection measures were implemented as required to ensure optimal crop health.

The crop was harvested once over 80% of the plants exhibited leaf senescence, indicating they had reached physiological maturity. Following harvest, tubers underwent a curing process to enhance their protective layers and durability. This was achieved by storing the tubers in a shaded and ventilated environment with 85% relative humidity and 15°C–20°C temperature for 2 weeks. The curing process increased tuber thickness and hardness, thereby improving resistance to diseases and pests. Before storage, tubers were screened to eliminate broken, diseased, or off‐type specimens. The selected tubers were placed on ventilated racks with nets, exposed to diffused light, and stored at ambient room temperature to evaluate their storability. This meticulous procedure ensured that the stored tubers maintained high quality and reduced postharvest losses.

### 2.4 Data Management and Parameter Estimation

Data on plant height (cm), stem number per hill, tuber number per hill, tuber weight per hill (kg), tuber yield (t ha^−1^), dry matter (%), tuber grading (mm), storability, weight loss (WL), losses resulting from rot (RL), as well as disease and insect infestation were recorded during the experimental period.

To record the data on plant height (cm), 10 randomly chosen plants from each plot were measured for height on a meter scale from ground level to the top of the plants. The average height of the plants was then calculated in centimeters (cm). The plant height was recorded 60 days after planting from the inner row of each plot.

In the case of data on stem number per hill, 10 plants were randomly selected from each plot, and their stems were counted. The average stem count per hill was then determined at 60 days post‐planting (DAP).

Tubers from 10 randomly selected plants within the net plot area were counted to know the tuber number per hill, and the mean number of tubers per plant or hill was then calculated (Abera et al. 2019).

Similarly, the tuber weight from 10 randomly selected plants in each plot was measured by using a digital weighing scale to record the tuber weight per hill (kg), and the average weight per hill was then calculated.

Besides these above data, the tuber yield from the entire plot was measured at 95 days after planting (DAP) and converted to t ha^−1^.

(1) $$\text{Tuber yield}\;\left(t\;{\mathrm{ha}}^{-1}\right)=\frac{\text{Total tuber weight}\;\left(\mathrm{kg}\right)\times 1000}{\text{Harvested area}\;\left(m^{2}\right)\times 10,000}$$

The dry matter percentage of tubers, a critical indicator of potato processing quality (Leonel et al. 2017), was determined using a standardized procedure. Five whole tubers were randomly selected from each plot and cut into small slices measuring 1–2 mm. These slices were thoroughly mixed and pre‐dried under sunlight for 2–3 days to reduce moisture content. The samples were then placed in a drying oven (Memmert GmbH, UN‐260, Germany) set at 70°C for 72 h to obtain their dry weight, ensuring accurate dry matter content analysis. The subsequent equation was employed to ascertain the DM content:

(2) $$\mathrm{Dry}\;\text{matter}\;\left(\%\right)=\frac{\mathrm{Dry}\;\text{weight}}{\text{Fresh weight}}\times 100$$

The process of grading plays a vital role and significantly impacts customers' choices when they are selecting a cultivar. The weight‐based grading of potato tubers is a significant attribute used to determine the suitability of a variety for processing and export (Kundu et al. 2022). The tubers harvested from each plot were classified based on their diameter into three categories: < 40, 41–55, and > 55 mm. They were then sorted and weighed accordingly (Huda et al. 2021). The average mean of tuber grading (%) by weight was computed over four locations.

To know the storability of potatoes, approximately 5 kg freshly, healthy, clean, and uniformly sized tubers were harvested for each variety and then were placed on ventilated netted racks and stored under diffused light at room temperature. The storage trial, conducted over 3 months (March–May), followed a randomized complete block design (RCBD) with three replications to assess shelf life (Pande and Luthra 2003). The initial weights of each variety were recorded, and the tubers were weighed at 15‐day intervals for a period of 90 days. The calculation of weight loss involved subtracting the weights of tubers at regular intervals from the starting weight during storage time. The weight loss was then reported as a percentage by the following formula (Upadhyay et al. 2020):

(3) $$\%\mathrm{WL}=\frac{\mathrm{IW}-\mathrm{FW}}{\mathrm{IW}}\times 100$$

where %WL = percentage total weight loss, IW = initial tuber weight (kg), and FW = final tuber weight (kg).

Tubers infected with Fusarium dry rot (FDR) or bacterial soft rot (BSR) were weighed prior to disposal. The losses resulting from rot were calculated using the following formula:

(4) $$\mathrm{RL}\;\left(\%\right)=\frac{\text{Total number of rotted tuber}\left(\mathrm{due}\;\text{to}\;\mathrm{FDR}\;\text{or}\;\mathrm{BSR}\right)}{\text{total tuber number}}\times 100$$

The tubers were laid out on the floor, and diseased tubers (affected by soft rot, dry rot, and common scab) were separated and weighed for each bag. The total number of tubers and the number of infected tubers were recorded to determine the disease incidence (Dey et al. 2010; Rahman et al. 2021).

The disease data on potato was taken from the field condition. The disease reactions were determined using the following formula:

(5) $$\begin{array}{cc} \text{Disease incidence}\;(\%)=\; & \left(\text{Infected plants}\;(\mathrm{no}.\right)\\ & ÷\text{Total plants in the plot}\;(\mathrm{no}.))\times 100 \end{array}$$

(6) $$\begin{array}{cc} \text{Common scab incidence}\;(\%)=\; & (\text{Infected tubers}\;\mathrm{by}\;\text{scab}\\ & ÷\text{Total harvested tubers})\times 100 \end{array}$$

(7) $$\begin{array}{cc} \mathrm{Cut}\;\text{worm infestation}\;(\%)=\; & (\text{Infested tubers}\;\mathrm{by}\;\text{cutworms}\\ & ÷\text{Total harvested tubers})\times 100 \end{array}$$

### 2.5 Estimation of Variance Components and Heritability

Variance components for each trait were estimated using a linear mixed model fitted via Restricted Maximum Likelihood (REML). In the model, genotype was treated as a random effect, while environment (combination of location and year) was considered a fixed effect. The genotype × environment interaction was also modeled as a random effect. Heritability on a genotype‐mean basis was calculated as:

$$h^{2}=\frac{\sigma_{G}^{2}}{\sigma_{G}^{2}+\frac{\sigma_{\mathrm{GE}}^{2}}{e}+\frac{\sigma_{\epsilon }^{2}}{\mathrm{re}}}$$

where $\sigma_{G}^{2}$ is the genotypic variance, $\sigma_{\mathrm{GE}}^{2}$ is the genotype × environment variance, $\sigma_{\epsilon }^{2}$ is the residual variance, e is the number of environments, and r is the number of replications. Spatial corrections were not applied, as trials were conducted using a randomized complete block design with appropriate replication.

### 2.6 MGIDI Workflow

All trait values were standardized (mean = 0, standard deviation = 1) to ensure comparability across different measurement scales. Factor analysis was performed on the correlation matrix of standardized traits using the Kaiser criterion (eigenvalues > 1) to extract factors. A varimax rotation was applied to improve factor interpretability. Each trait was then linked to the factor on which it had the highest loading after rotation. The MGIDI index for each genotype was calculated as the Euclidean distance between the genotype's factor scores and the ideal genotype (ideotype), which represents the most desirable value (maximum or minimum, depending on trait direction) for all traits. The MGIDI is formally expressed as:

$$\text{MGID}I_{i}=\sqrt{\sum_{j=1}^{f}{\left(\gamma_{\mathrm{ij}-}\gamma_{j}^{\text{ideal}}\right)}^{2}}$$

where $\gamma_{\mathrm{ij}}$ is the score of genotypes *i* on factor *j*, and $\gamma_{j}^{\text{ideal}}$ is the ideal (target) score for factor *j*. Genotypes with lower MGIDI values are closer to the ideotype and are thus considered more desirable.

### 2.7 Stability Analysis

Stability of genotype performance across environments was assessed using two approaches:

1. GGE biplot analysis was conducted based on the site regression (SREG) model, which combines genotype main effect (G) and genotype × environment interaction (GE). Principal components (PC1 and PC2) were extracted via singular value decomposition (SVD) of the G + GE matrix. Genotypes with low PC2 scores and short projections from the average environment axis were considered stable.
2. Regression based stability was assessed by calculating the regression coefficient ($b_{i}$) of each genotype across environmental indices, along with the deviation from regression $\left(S_{d}^{2}\right)$. Genotypes with $b_{i}\approx 1$ and low $S_{d}^{2}$ values were considered stable and widely adapted.

### 2.8 Data Analysis

The statistical analysis was performed using the R software v4.2.0 (R Core Team 2013). The “metan” package in R was utilized for data manipulation and the computation of the MGIDI (Olivoto and Nardino 2021). Data visualization, including bar graphs, boxplots, and Principal Component Analysis (PCA), was carried out using the “ggplot2” package in R.

## 3 Results

### 3.1 Selection of Genotypes Using Multitrait Genotype Ideotype Distance (MGIDI) Index

**FIGURE 3:**
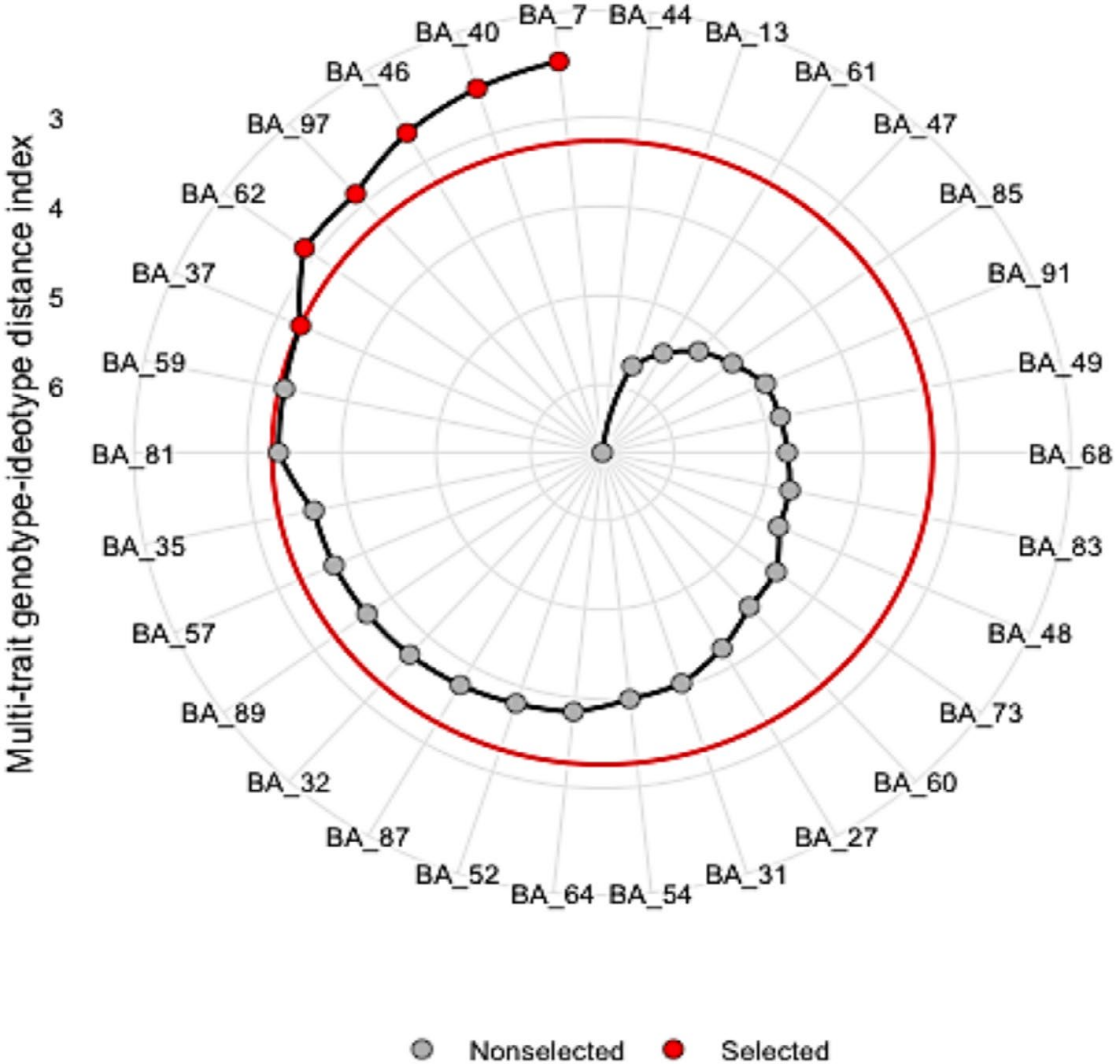
Ranking and selection of potato varieties using the MGIDI index. Genotypes selected according to the index are represented by red dots, while nonselected genotypes are indicated by ash‐colored dots. The central red circle denotes the selection threshold, determined by a 15% selection intensity.

The MGIDI index, a multivariate selection tool, was employed to identify superior potato genotypes aligned with the ideotype. Based on this index, six potato varieties—BARI Alu‐7, BARI Alu‐40, BARI Alu‐46, BARI Alu‐97, BARI Alu‐62, and BARI Alu‐37—were selected as the top‐performing genotypes, considering tuber yield and other agronomic traits (Figure 3). Among these, BARI Alu‐7 ranked highest in terms of proximity to the ideotype. The variance components and genetic parameters of the evaluated traits are detailed in Table S2.

The qualities retained by the top four principal components (PCs) explained 89.2% of the total variation (Table 2). Using eigenvalues (> 1), the MGIDI index identified four factors (Table 3) that captured a significant portion of the variance for each trait. Factor 1 was associated with tuber weight per hill (TWH) and yield at 95 days after planting (Y95), while factor 2 correlated with stem number (SN), total number of tubers per hill (TNH), and grade (> 55). Factor 3 was linked to plant height (PH) and dry matter content (DM), and factor 4 was related to grade (41–55). This dimensionality reduction preserved high explanatory power, with an average communality of 0.89 (ranging from 0.7 to 0.97).

**TABLE 2:** Principal components, eigenvalues, explained variance, and cumulative variance obtained in the factor analysis.

Principal components	Eigenvalues	Variance	Cumulative variance (%)
PC1	2.86	35.8	35.8
PC2	1.91	23.8	59.6
PC3	1.34	16.8	76.4
PC4	1.02	12.8	89.2
PC5	0.49	6.08	95.3
PC6	0.33	4.18	99.4
PC7	0.04	0.55	100

**TABLE 3:** Communalities and uniqueness of traits derived from MGIDI factor analysis.

Traits	FA1	FA2	FA3	FA4	TC	TU
PH	0.43	0	0.78	0.14	0.81	0.19
SN	0.39	0.56	−0.3	−0.38	0.70	0.3
TNH	0.39	0.81	0.28	0.29	0.96	0.04
TWH	0.97	0.04	0.11	0.05	0.97	0.03
Y95	0.98	0.04	0.11	0.05	0.97	0.03
DM	−0.04	0	0.92	−0.11	0.86	0.14
G (41–55)	−0.09	0.08	0.05	−0.96	0.93	0.07
G (> 55)	0.25	−0.91	0.07	0.22	0.95	0.05

Abbreviations: DM, dry matter; FA, factor; G (41–55), tuber grade of 41–55 mm; G (> 55), tuber grade of > 55 mm; PH, plant height; SN, stem number; TC, trait communality; TNH, tuber number per hill; TU, trait uniqueness; TWH, tuber weight per hill; Y95, tuber yield at 95 days after planting.

**FIGURE 4:**
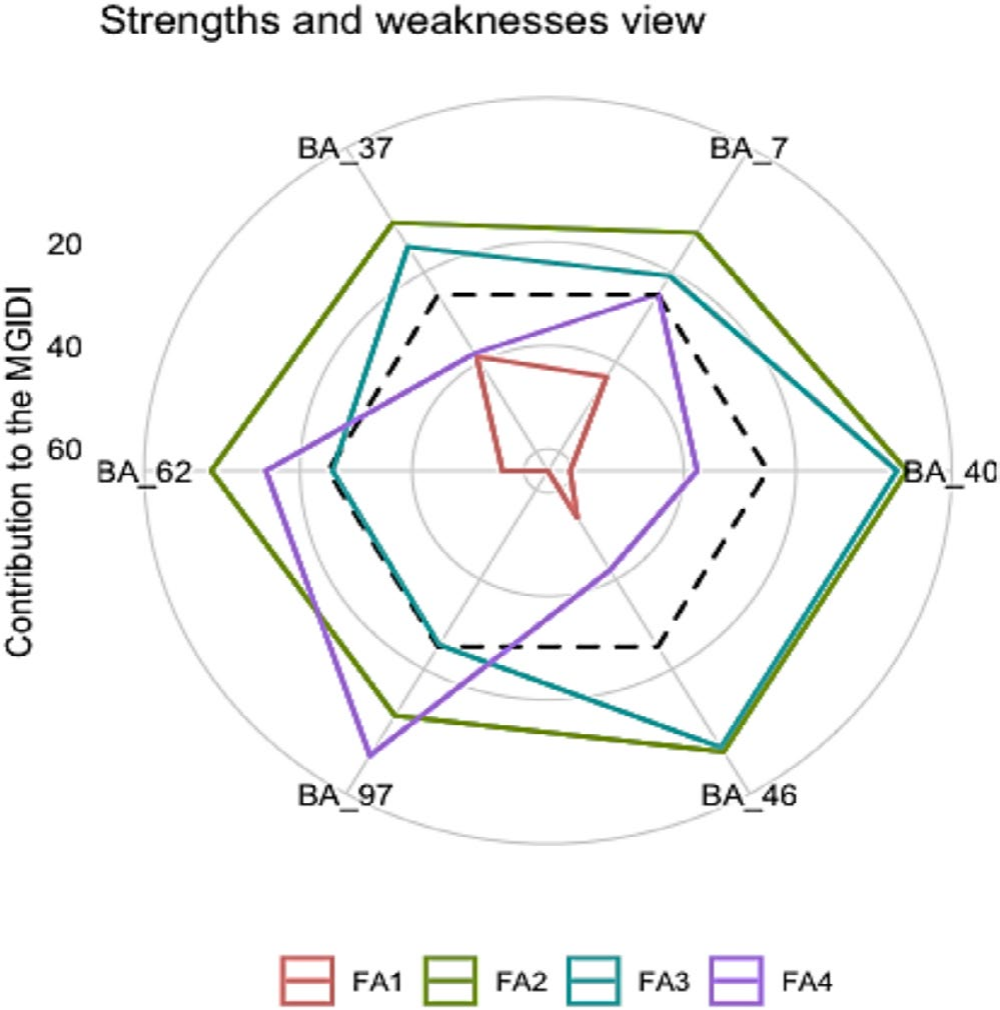
Strengths and weaknesses view of the selected genotypes where the dotted line indicates the theoretical value.

The MGIDI index's ability to decompose the contribution of each factor aids in identifying genotype strengths and weaknesses. Each factor's contribution is ranked, with factors closer to the center of the plot making the higher factor values and less contributions, while those near the edges exhibit lower factor values serve as an indication of strength. The dashed line in Figure 4 represents the theoretical scenario where all factors contribute equally to the MGIDI index, providing a benchmark for comparison. Figure 4 illustrates the relative contributions of factors for different genotypes. Factor 1 (FA1) made the smallest contribution to the MGIDI index for BARI Alu‐97, while BARI Alu‐37 exhibited the largest contribution. However, all the genotypes showed much lower performance than average value in case of factor 1. BARI Alu‐62 and BARI Alu‐97 showed their strength in factors 3 and 4, and these two factors are important for export potential genotype selection.

### 3.2 Export Potential Evaluation of the Selected Genotypes

#### 3.2.1 Yield Stability

**FIGURE 5:**
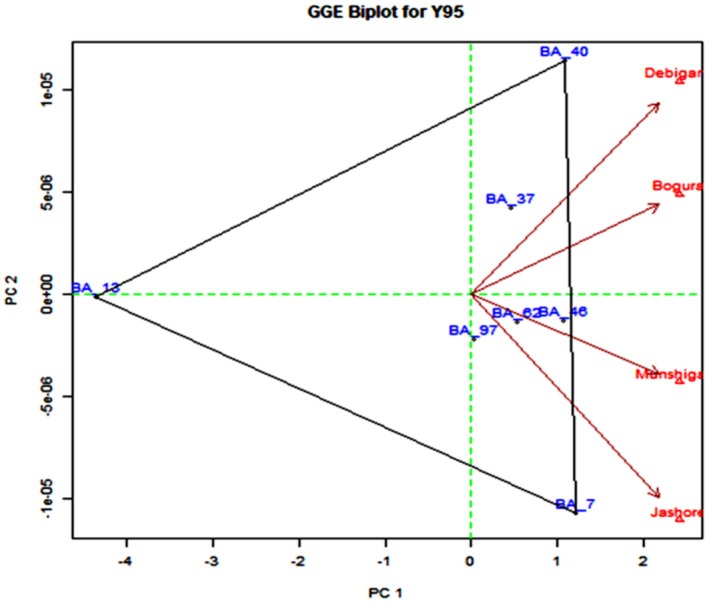
Stability of the varieties (principal component 1 vs. principal component 2) over the environment.

Genotypes with PC1 scores greater than zero were classified as high‐yielding and stable, while those with PC1 scores less than zero were categorized as low yielding and unstable. Figure 5 highlights that BARI Alu‐37, BARI Alu‐40, BARI Alu‐46, BARI Alu‐62, BARI Alu‐7, and BARI Alu‐97 demonstrated superior performance, whereas BARI Alu‐13 (used as a check variety) was identified as low yielding and unstable. The yields of BARI Alu‐37, BARI Alu‐40, BARI Alu‐46, BARI Alu‐62, BARI Alu‐7, and BARI Alu‐97 were statistically nonsignificant (Table 4).

**TABLE 4:** Yield, regression coefficient (*b*_*i*_), deviation from regression (*s* ^2^ *d*_*i*_) of the selected potato genotypes.

Genotypes	Mean yield	Regression coefficient (*b*_*i*_)	Deviation from regression (*s* ^2^ *d*_*i*_)
BARI Alu‐13	26.02 ± 2.28b	0.95	2.28
BARI Alu‐37	37.61 ± 3.06a	1.22	3.55
BARI Alu‐40	39.13 ± 3.20a	1.54	8.50
BARI Alu‐46	39.07 ± 2.37a	0.64	1.65
BARI Alu‐62	37.78 ± 2.41a	0.92	0.53
BARI Alu‐7	39.40 ± 2.97a	0.64	9.11
BARI Alu‐97	36.57 ± 2.27a	1.07	1.78

*Note:* Values in the same column with different letters represent statistically significant differences, whereas those with the same letter indicate no significant difference at the 5% significance level.

Among the genotypes, BARI Alu‐46 and BARI Alu‐62 achieved mean yields of 39.07 and 37.78 t ha^−1^, respectively, with regression coefficients of 0.642 and 0.92 and deviations from regression of 1.658 and 0.538. These parameters indicate their stability and predictability. Similarly, BARI Alu‐7 and BARI Alu‐37 also met the criteria for stability, reinforcing their potential as reliable high‐yielding genotypes.

#### 3.2.2 Dry Matter, Tuber Grading, and Diseases Incidence

The dry matter concentration of potatoes is a critical quality attribute, particularly for processing purposes. Potatoes cultivated in Bangladesh typically exhibit lower dry matter content due to climatic conditions and traditional farming practices. For processed potato products, varieties with a dry matter concentration of 20% or higher are preferred. Significant variations in dry matter content were observed among the evaluated potato genotypes (Table 5). Notably, BARI Alu‐40 and BARI Alu‐46 demonstrated the highest dry matter concentrations, at 21.90% and 21.88%, respectively, across all locations. Overall, the dry matter percentage ranged between 18% and 22%.

**TABLE 5:** Dry matter, tuber grading, and pest and disease incidence of the selected potato genotypes.

Genotypes	Dry matter (%)	Tuber grade over A grade (41–55) mm	Scab incidence (%)	Crack incidence (%)	Cutworm incidence (%)
BARI Alu‐13	18.95 ± 0.61c	53.21 ± 3.86b	0.00	1.52	0.87
BARI Alu‐37	20.17 ± 0.48bc	53.42 ± 4.25b	0.65	0.00	0.81
BARI Alu‐40	21.90 ± 0.62a	56.18 ± 3.66ab	0.45	1.81	0.63
BARI Alu‐46	21.88 ± 0.67a	55.09 ± 3.21ab	0.00	0.53	1.88
BARI Alu‐62	19.77 ± 0.45c	61.43 ± 3.51a	0.00	0.00	1.77
BARI Alu‐7	21.32 ± 0.44ab	55.07 ± 1.80ab	7.05	0.95	0.95
BARI Alu‐97	20.42 ± 0.31abc	62.73 ± 2.36a	0.00	0.50	0.79

**FIGURE 6:**
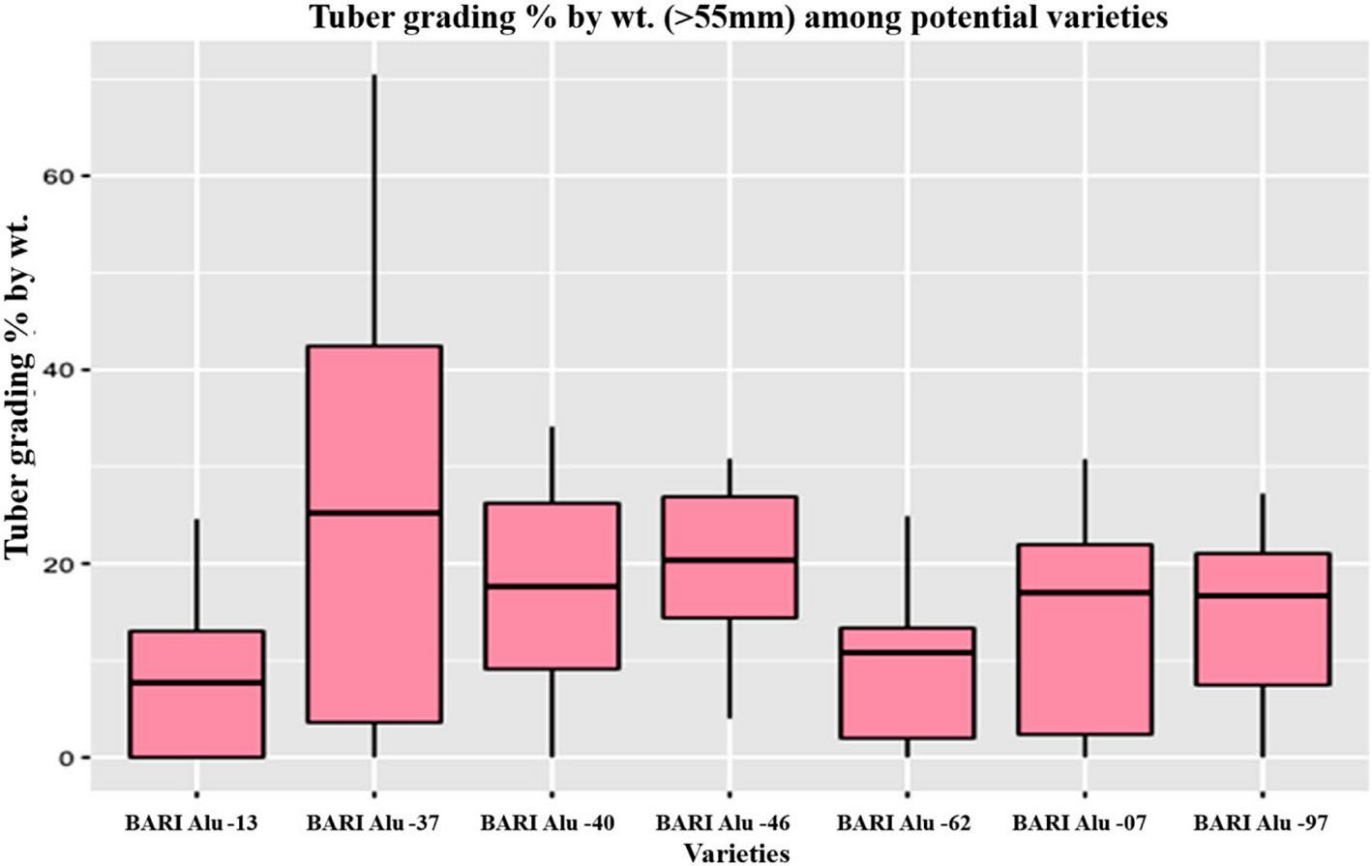
Variation of oversized tuber (%) among potato cultivars.

In terms of marketability, grading and sorting play a vital role in meeting quality standards, ensuring that buyers receive consistent products, and optimizing the suitability for export markets. Larger tubers are generally favored in international markets, as smaller tubers are less desirable for export. Among the genotypes, BARI Alu‐97 and BARI Alu‐62 produced the highest proportion of 41–55 mm sized tubers accounting for 62.73% and 61.43%, respectively (Table 5). On the other hand, BARI Alu‐37 exhibited the highest proportion (25.35%) of tubers exceeding 55 mm in size (Figure 6), followed by BARI Alu‐40, BARI Alu‐46, and BARI Alu‐7. However, specific genotypes faced notable challenges: scab was a significant issue in BARI Alu‐7, while crack damage was prevalent in BARI Alu‐40. Additionally, BARI Alu‐46 recorded the highest incidence of cutworm damage, followed by BARI Alu‐62 (Table 5).

#### 3.2.3 Storability

Significant differences were observed among the potato varieties in terms of cumulative weight loss and rotting loss under natural storage conditions (Tables 6 and 7). After 90 days of storage, BARI Alu‐62 and BARI Alu‐97 exhibited the lowest weight losses, recorded at 14.08% and 18.32%, respectively. A similar trend was noted for cumulative rotting losses caused by bacterial soft rot and Fusarium dry rot. During the 2020–2021 storage period, BARI Alu‐62 demonstrated exceptional resistance, with no recorded rotting loss due to bacterial soft rot or Fusarium dry rot up to 90 days in natural storage conditions.

**TABLE 6:** Cumulative weight loss (%) of potato cultivars on different days after storage.

Year	Variety/Genotype	Cumulative weight loss (%) at different DAS					
		15 days	30 days	45 days	60 days	75 days	90 days
2019–2020	BARI Alu‐13	5.47	6.22	14.67	15.12	19.43	38.75
	BARI Alu‐37	2.89	4.88	16.07	17.19	28.47	30.11
	BARI Alu‐40	5.84	11.92	15.24	28.94	33.60	37.14
	BARI Alu‐46	6.32	8.89	12.15	22.06	25.82	29.58
	BARI Alu‐62	5.70	7.03	10.26	11.74	12.76	14.21
	BARI Alu‐7	3.73	7.88	23.50	24.30	27.83	31.00
	BARI Alu‐97	4.53	8.89	14.03	16.10	18.48	21.30
2020–2021	BARI Alu‐13	2.52	3.07	4.24	5.08	8.49	33.93
	BARI Alu‐37	3.26	3.50	5.47	10.64	14.11	19.92
	BARI Alu‐40	7.38	8.49	10.40	17.64	21.36	30.55
	BARI Alu‐46	1.18	2.42	4.54	6.17	9.40	11.93
	BARI Alu‐62	3.96	4.90	5.83	7.09	8.33	13.95
	BARI Alu‐7	2.41	7.76	28.47	28.91	30.63	33.12
	BARI Alu‐97	5.34	6.79	9.88	12.21	12.96	15.33
Mean	BARI Alu‐13	3.99 ± 1.48	4.64 ± 1.58	9.45 ± 5.22	10.10 ± 5.02	13.96 ± 5.47	36.34 ± 2.41
	BARI Alu‐37	3.08 ± 0.19	4.19 ± 0.69	10.77 ± 5.30	13.92 ± 3.28	21.29 ± 7.18	25.01 ± 5.10
	BARI Alu‐40	6.61 ± 0.77	10.21 ± 1.72	12.82 ± 2.42	23.29 ± 5.65	27.48 ± 6.12	33.85 ± 3.30
	BARI Alu‐46	3.75 ± 2.57	5.66 ± 3.24	8.34 ± 3.81	14.11 ± 9.95	17.61 ± 8.21	20.76 ± 8.83
	BARI Alu‐62	4.83 ± 0.87	5.96 ± 1.07	8.05 ± 2.22	9.41 ± 2.33	10.55 ± 2.22	14.08 ± 0.13
	BARI Alu‐7	3.07 ± 0.66	7.82 ± 0.06	25.99 ± 2.49	26.61 ± 2.70	29.23 ± 1.40	32.06 ± 1.06
	BARI Alu‐97	4.94 ± 0.41	7.84 ± 1.05	11.95 ± 2.08	14.16 ± 1.95	15.72 ± 2.76	18.32 ± 2.99

Abbreviation: DAS, days after storage.

**TABLE 7:** Cumulative rotting loss (%) of potato cultivars due to bacterial soft rot/Fusarium dry rot on different days after storage.

Year	Variety/Genotype	Cumulative rotting loss (%) at different DAS					
		15 days	30 days	45 days	60 days	75 days	90 days
2019–2020	BARI Alu‐13	0.00	2.35	4.71	7.06	10.59	17.18
	BARI Alu‐37	0.68	0.68	6.80	8.16	12.24	16.33
	BARI Alu‐40	0.00	4.81	5.77	6.73	13.46	29.81
	BARI Alu‐46	0.00	1.14	2.25	8.99	10.19	12.30
	BARI Alu‐62	0.84	0.84	1.71	5.88	7.56	8.40
	BARI Alu‐7	0.00	1.15	4.60	8.05	9.20	17.64
	BARI Alu‐97	0.00	4.35	5.87	8.65	10.61	12.26
2020–2021	BARI Alu‐13	0.00	1.56	1.56	1.56	1.56	4.69
	BARI Alu‐37	1.47	5.88	5.88	5.88	8.82	10.29
	BARI Alu‐40	1.45	5.80	5.80	5.80	11.59	13.04
	BARI Alu‐46	0.00	3.39	3.39	5.08	5.08	6.78
	BARI Alu‐62	0.00	0.00	0.00	0.00	0.00	0.00
	BARI Alu‐7	2.78	5.56	8.33	10.64	12.33	15.33
	BARI Alu‐97	0.00	0.00	5.97	5.97	5.97	5.97
Mean	BARI Alu‐13	0.00 ± 0.00	1.96 ± 0.40	3.13 ± 1.58	4.31 ± 2.75	6.08 ± 4.52	10.93 ± 6.25
	BARI Alu‐37	1.08 ± 0.40	3.28 ± 2.60	6.34 ± 0.46	7.02 ± 1.14	10.53 ± 1.71	13.31 ± 3.02
	BARI Alu‐40	0.72 ± 0.73	5.30 ± 0.50	5.78 ± 0.02	6.26 ± 0.47	12.53 ± 0.94	21.43 ± 8.39
	BARI Alu‐46	0.00 ± 0.00	2.26 ± 1.13	2.82 ± 0.57	7.04 ± 1.96	7.64 ± 2.56	9.54 ± 2.76
	BARI Alu‐62	0.42 ± 0.42	0.42 ± 0.42	0.85 ± 0.86	2.94 ± 1.14	3.78 ± 1.20	4.20 ± 1.23
	BARI Alu‐7	1.39 ± 1.39	3.35 ± 2.21	6.46 ± 1.87	9.34 ± 1.30	10.76 ± 1.57	16.49 ± 1.16
	BARI Alu‐97	0.00 ± 0.00	2.17 ± 1.10	5.92 ± 0.05	7.31 ± 1.34	8.29 ± 2.32	9.12 ± 3.15

Abbreviation: DAS, days after storage.

## 4 Discussion

Breeders frequently aim to merge multiple desirable traits into a novel genotype to achieve superior performance. Choosing a genotype from the ideotypes might be challenging when assessing many traits. Regarding this matter, several multivariate techniques are commonly employed, including principal component analysis, factor analysis, cluster analysis, and various sampling approaches to categorize measurable traits or choose test genotypes (Bhandari et al. 2017). In this work, we employed PCA to establish a connection between the genotypes of the tests and the measured traits. The MGIDI is a novel tool that facilitates the identification of genotypes with multiple desirable characteristics. The use of MGIDI for genotype selection is advantageous due to its ability to address multicollinearity difficulties, achieve a high success rate in selecting traits with desirable gains, and identify the strengths and weaknesses of genotypes (Olivoto and Nardino 2021). To use this index, factor analysis must be applied. Factor analysis is a powerful technique that represents observable genotypes using unobserved latent components. It achieves this by maximizing the shared variance across correlated genotypes (Momen et al. 2021). In our study, we evaluated morphological characters such as plant height, stem per hill, tuber number per hill, tuber weight per hill, yield at 95 days, and tuber grading. The MGIDI was used to assess the export potentiality of a collection of white‐skinned potato genotypes which identified BARI Alu‐7, BARI Alu‐40, BARI Alu‐46, BARI Alu‐97, BARI Alu‐62, and BARI Alu‐37 as high‐performing genotypes in both growing seasons. In a study of 130 eggplant genotype evaluations, four eggplant genotypes were selected through the MGIDI approach (Uddin et al. 2021). The MGIDI index was used to identify leaf blast‐resistant rice genotypes (Jalalifar et al. 2023). They evaluated several resistance and susceptibility indices of rice leaves and ultimately selected 17 genotypes out of the total 153 investigated. The genotype at the cut‐off point of the MGIDI index plays a critical role in breeding performance (Tigist et al. 2013). In this study, BARI Alu‐59 is in the cut‐off point (Figure 3), which indicates the better possibility of this genotype in breeding performance. Therefore, the researcher should give special consideration to evaluating genotypes that are near the threshold.

The favorable climate and soil conditions of Bangladesh make it an ideal location for growing potatoes at competitive prices, primarily due to the relatively low cost of labor compared to other potato‐producing countries. In our study, we selected 30 white‐skinned genotypes for evaluation with international consumers in mind. Based on key attributes such as tuber yield, dry matter content, tuber grading, storability, and disease resistance, BARI Alu‐62 and BARI Alu‐97 have emerged as the top choices among the six selected varieties for producing export‐quality potatoes. Essential factors for export suitability include stem number per hill, tuber yield, dry matter content, tuber grading, storability, and disease incidence. Farmers often use intercropping strategies with potatoes and pumpkins on the same land, where fewer stems per hill are preferred to allow pumpkin growth. Additionally, focusing on tuber size and yield with minimal input is crucial. Customers prefer potatoes with minimal peeling loss; small‐sized tubers with higher peeling losses are less desirable. Storability is another essential trait, as longer lasting tubers are better suited for transport and extended storage. Disease incidence negatively affects tuber appearance and consumer appeal.

Research by Girma et al. (2004) and Chala and Dechasa (2015) emphasize the significance of high yield and quick maturation in potato export varieties, which aligns with our findings that BARI Alu‐62 and BARI Alu‐97 possess these advantageous traits. Our research identified that BARI Alu‐97 produces more than 80% tuber belonging to 41–55 and > 55 mm grade which is desired for export criteria. Studies by Galdón et al. (2012) and Gibson and Kurilich (2013) mentioned the importance of nutritional content and tuber quality. Our study corroborates these findings by focusing on dry matter content, storability, and disease resistance as critical factors. Yue et al. (2022) demonstrated that evaluating traits relevant to environmental conditions can identify genotypes well‐adapted to specific environments. Similarly, Debsharma et al. (2022) and Singamsetti et al. (2023) highlighted the importance of multitrait selection in meeting quality standards of the crops.

Identifying varieties that yield the highest percentage of premium‐grade tubers is essential for expanding market opportunities. The identification of BARI Alu‐62 and BARI Alu‐97 as leading export varieties is a significant outcome of our research. Despite their strong overall performance, both BARI Alu‐62 and BARI Alu‐97 exhibited minor trade‐offs that are worth noting. BARI Alu‐62 showed a relatively higher incidence of cutworm damage (1.77%) compared to other genotypes, although it was free from scab and cracking and had an excellent proportion of export‐grade tubers (61.43% in 41–55 mm). BARI Alu‐97 had the highest proportion of export‐grade tubers (62.73%) and an acceptable dry matter level, with only minimal cutworm presence (0.79%) and negligible cracking. These issues were not consistent across locations and were considered manageable based on consultations with exporters. From a practical export chain perspective, these genotypes still meet key requirements, and their advantages outweigh the minor drawbacks, making them viable candidates for large‐scale adoption.

In addition to trait stability and market suitability, postharvest storability remains a critical factor for export‐oriented cultivars. Potato breeders must also develop breeding strategies to produce special varieties or cultivation packages capable of generating the highest proportion of premium‐grade tubers. Another important factor that exporters consistently look for is varieties that have a long storage life. Prolonged dormancy is crucial for international shipping, as potatoes must remain undamaged upon arrival at their destinations. Previous research indicated that moisture loss during storage due to respiration and transpiration significantly affects potato quality (Tigist et al. 2013). We observed that BARI Alu‐62 can be stored for 3 months without deformities, cracks, water loss, or shrinkage, which indicates it is a good keeper and suitable for export, as supported by Luthra et al. (2006). This finding is critical as disease‐free tubers significantly enhance marketability and consumer acceptance, reducing risks for exporters and improving profitability (Islam, Naznin, et al. 2022; Luthra et al. 2006). Another study conducted by Upadhyay et al. (2020) discovered that the genotypes Yagana (12.3%) and L‐235.4 (14.7%) exhibited much lower weight loss after 120 days of storage at room temperature, followed by K. Jyoti (16.1%) and Khumal Seto‐1 (16.5%). Higher dry matter content is indicative of better processing quality, as it reduces water content and increases starch concentration (Leonel et al. 2017; Baranowska 2019). Low dry matter‐containing tubers are less qualified for industrial processing (Bedini et al. 2023). We observed differences in dry matter content among the varieties in this investigation. Dry matter ranged from 19% to 22%, which is considered favorable under Bangladeshi agroecological conditions. Environmental and genetics impact dry matter content in potato (Miller et al. 1975; Tai and Coleman 1999). According to Rastovski et al. (1981) hereditary traits govern dry matter, but it can also be influenced by water intake, temperature, photoperiod, and other factors.

Beyond agronomic performance, the integration of BARI Alu‐62 and BARI Alu‐97 into Bangladesh's potato export system appears practically feasible. These genotypes meet the size, dry matter, and storage preferences of key export markets such as Malaysia, Sri Lanka, Singapore, and the Middle East. Both are compatible with the country's existing cold chain infrastructure, which typically allows 90–120 days of storage in private facilities. Additionally, as officially released varieties by BARI, their seed multiplication is underway through both public and private channels, ensuring scalability. These alignments make BARI Alu‐62 and BARI Alu‐97 strong candidates not just agronomically, but logistically, for large‐scale export‐oriented cultivation.

The results of this study have significant implications for both breeding practices and policy development in Bangladesh's potato industry. Genotypes such as BARI Alu‐62 and BARI Alu‐97 can help breeders align production strategies with global market requirements, thereby improving the export potential of Bangladeshi potatoes. However, despite the robustness of the MGIDI‐based evaluation, several important limitations should be acknowledged. Key processing‐quality traits such as reducing sugar levels, sugar accumulation during storage, fry color, and oil absorption were not assessed, yet these are critical for processed potato products and directly affect acceptance in consumer markets (Bedini et al. 2023). Similarly, consumer‐oriented traits, including peeling efficiency, flavor, and texture, were not considered, although they play a role in overall marketability and satisfaction (Bedini et al. 2023). Moreover, the study did not include economic metrics or postharvest handling simulations under variable temperature and humidity conditions, which are known to affect tuber weight loss, sprouting, and disease risk during transit and storage (Debsharma et al. 2022). Future research addressing these gaps would enhance the evaluation of export potential. Future studies should integrate molecular breeding approaches, expand the MGIDI framework to include consumer preferences and economic feasibility, and explore advanced postharvest technologies like controlled atmosphere storage to reduce losses and extend the shelf life of export‐grade potatoes.

## 5 Conclusion

This study identified BARI Alu‐37, BARI Alu‐40, BARI Alu‐46, BARI Alu‐62, BARI Alu‐7, and BARI Alu‐97 as high‐performing potato genotypes based on the MGIDI. Among these, BARI Alu‐62 and BARI Alu‐97 are recommended for export due to their superior performance in yield stability, dry matter content, tuber size, storability, and disease resistance. These findings highlight the potential to enhance Bangladesh's potato export industry through targeted breeding programs and improved postharvest practices. Future efforts should focus on integrating advanced breeding techniques and addressing consumer preferences to further improve export‐quality potato production.

## Author Contributions

M.M.I., M.N.A., M.M.R., S.N., M.N.U., A.N., T.H., M.S., M.B.A.: conceptualization and methodology; M.M.I., A.A., A.G., M.M.A., A.H.: data collection and analysis; M.M.I., M.N.A., M.M.R., S.N., M.N.U., A.N., T.H., M.S., M.B.A.: writing the manuscript; M.N.A., A.A., A.G., M.M.A., A.H.: reviewing and final editing; M.N.A., A.A., A.G., M.M.A., A.H.: supervision; M.M.I., M.N.A., A.A., A.G., M.M.A., A.H.: funding and project administrator. All authors reviewed the findings and accepted the final version of the manuscript.

## Funding

This research was financially supported by the Bangladesh Agricultural Research Institute, Gazipur, Bangladesh, and the Bangladesh Wheat and Maize Research Institute, Dinajpur, Bangladesh. This research was also funded by the Deanship of Graduate Studies and Scientific Research, Taif University, Taif, Saudi Arabia.

## Ethics Statement

The authors have nothing to report.

## Consent

The authors have nothing to report.

## Conflicts of Interest

The authors declare no conflicts of interest.

## Supporting information


**Table S1:** Tuber characteristics of 30 BARI released potato cultivars.
**Table S2:** Deviance analysis, estimated variance components, and genetic parameters for eight agronomic traits of 30 potato genotypes.

## Data Availability

The datasets generated during and/or analyzed during the current study will be available from the corresponding author upon request.
