# Supplementary material for: Selection of Potential Potato Cultivars With Export Quality Through Multitrait Genotype‐Ideotype Distance Index (MGIDI)
Source: Food Sci Nutr. 2026 Feb 10;14(2):e71524. doi: 10.1002/fsn3.71524 (PMC12890580; doi:10.1002/fsn3.71524)
Supplement: Supplementary file 1 — Table S1: Tuber characteristics of 30 BARI released potato cultivars. Table S2: Deviance analysis, estimated variance components, and genetic parameters for eight agronomic traits of 30 potato genotypes. [file FSN3-14-e71524-s001.docx]

**Table S1**. Tuber characteristics of 30 BARI released potato cultivars

| Variety | Origin/  source | Release  Year | Colour base of flesh | General shape | Depth of eyes | Colour of skin | Texture of Skin | Colour of base of eye |
| --- | --- | --- | --- | --- | --- | --- | --- | --- |
| BARI Alu-13 | Netherland | 1994 | Yellow | Short-oval | Medium | Yellow | Partially netted | Yellow |
| BARI Alu-27 | Germany | 2008 | Dark yellow | Oval | Shallow | Yellow | Smooth | Yellow |
| BARI Alu-31 | Netherland | 2010 | Light yellow | Long-oval | Shallow | Yellow | Smooth | Yellow |
| BARI Alu-32 | Netherland | 2010 | Light yellow | Long-oval | Medium | Yellow | Smooth | Yellow |
| BARI Alu-35 | Bangladesh | 2012 | Light yellow | Short-oval | Deep | Yellow | Rough (flaky) | Yellow |
| BARI Alu-37 | Bangladesh | 2012 | Light yellow | Long-oval | Medium | Yellow | Smooth | Yellow |
| BARI Alu-38 | Germany | 2012 | Light yellow | Long-oval | Medium | Yellow | Partially netted | Yellow |
| BARI Alu-39 | Netherland | 2012 | Yellow | Oval | Shallow | Yellow | Partially netted | Yellow |
| BARI Alu-40 | Bangladesh | 2012 | Yellow | Long-oval | Medium | Yellow | Totally netted | Yellow |
| BARI Alu-44 | Netherland | 2012 | Light yellow | Long-oval | Medium | Yellow | Smooth | Yellow |
| BARI Alu-46 | CIP | 2013 | Cream | Oval | Medium | Yellow | Partially netted | Red |
| BARI Alu-47 | Bangladesh | 2014 | Light yellow | Long-oval | Medium | Yellow | Partially netted | Yellow |
| BARI Alu-49 | Bangladesh | 2014 | Light yellow | Short-oval | Medium | Yellow | Smooth | Yellow |
| BARI Alu-51 | Germany | 2014 | Yellow | Short-oval | Shallow | Pink | Smooth | Yellow |
| BARI Alu-52 | Netherland | 2014 | Light yellow | Short-oval | Shallow | Yellow | Smooth | Yellow |
| BARI Alu-54 | Netherland | 2014 | Yellow | Long-oval | Shallow | Yellow | Smooth | Yellow |
| BARI Alu-57 | Bangladesh | 2014 | Yellow-cream | Long-oval | Very Shallow | Yellow | Partially netted | Yellow |
| BARI Alu-59 | Netherland | 2014 | Yellow-cream | Long-oval | Shallow | Yellow | Partially netted | Yellow |
| BARI Alu-60 | Netherland | 2014 | Yellow-cream | Long-oval | Medium | Yellow | Smooth | Yellow |
| BARI Alu-61 | Netherland | 2014 | Yellow-cream | Oval | Medium | Yellow | Smooth | Yellow |
| BARI Alu-62 | Bangladesh | 2015 | Yellow-cream | Long-oval | Medium | Yellow | Partially netted | Yellow |
| BARI Alu-64 | Denmark | 2015 | Light yellow | Long-oval | Very Shallow | Yellow | Partially netted | Yellow |
| BARI Alu-68 | Netherland | 2016 | Yellow-cream | Short-oval | Medium | Yellow | Rough (flaky) | Yellow |
| BARI Alu-73 | CIP | 2017 | Yellow-cream | Oval | Medium | Yellow | Partially netted | Yellow |
| BARI Alu-81 | CIP | 2019 | Cream | Oval | Deep | Yellow | Partially netted | Yellow |
| BARI Alu-83 | Denmark | 2019 | Cream | Oval | Shallow | Yellow | Smooth | Yellow |
| BARI Alu-85 | Germany | 2019 | Cream | Long-oval | Very Shallow | Yellow | Smooth | Yellow |
| BARI Alu-86 | Bangladesh | 2019 | Yellow | Long | Very Shallow | Pink | Smooth | Yellow |
| BARI Alu-87 | CIP | 2019 | Yellow | Long | Very Shallow | Yellow | Rough (flaky) | Yellow |
| BARI Alu-89 | Netherland | 2019 | Dark yellow | Oval | Very Shallow | Yellow | Partially netted | Yellow |
| BARI Alu-91 | Netherland | 2019 | Yellow | Short-oval | Medium | White-cream | Partially netted | Red |
| BARI Alu-97 | Netherland | 2022 | Light yellow | Oval | Medium | Yellow | Smooth | Yellow |

**Table S2**. Deviance analysis, estimated variance components and genetic parameters for eight agronomic traits of thirty potato genotypes

| Factor | Parameters | Xo | Xs | SD | SDperc | h2 | SG | SGperc | sense | goal |
| --- | --- | --- | --- | --- | --- | --- | --- | --- | --- | --- |
| FA1 | TWH | 0.53 | 0.561 | 0.027 | 5.05 | 0.20 | 0.005 | 1.02 | increase | 100 |
| FA1 | Y95 | 35.60 | 37.4 | 1.8 | 5.05 | 0.20 | 0.365 | 1.03 | increase | 100 |
| FA2 | SN | 4.32 | 4.48 | 0.162 | 3.75 | 0.41 | 0.066 | 1.52 | increase | 100 |
| FA2 | TNH | 9.15 | 9.52 | 0.372 | 4.07 | 0.52 | 0.191 | 2.09 | increase | 100 |
| FA2 | G (>55) | 19.50 | 17.3 | -2.14 | -11 | 0.44 | -0.93 | -4.78 | increase | 0 |
| FA3 | PH | 60.90 | 63.4 | 2.47 | 4.06 | 0.20 | 0.485 | 0.79 | increase | 100 |
| FA3 | DM | 19.80 | 20.8 | 0.972 | 4.9 | 0.51 | 0.497 | 2.51 | increase | 100 |
| FA4 | G (41-55) | 53.00 | 54.6 | 1.62 | 3.06 | 0.07 | 0.113 | 0.21 | increase | 100 |

FA = Factor, TWH= Tuber weight per hill, Y95= Tuber yield at 95 days after planting, SN= Stem number, TNH= Tuber number per hill, G (>55) = Tuber grade of >55 mm, PH= Plant height, DM= Dry matter, G (41-55) = Tuber grade of 41-55 mm, Xo= Original mean, Xs=, SD= Selection differential, SDperc= , h2= Heritability, SG= Selection gain , SGperc= Selection Gain Percentage.
